# The abnormalities of free fatty acid metabolism in patients with hypertrophic cardiomyopathy, a single-center retrospective observational study

**DOI:** 10.1186/s12872-024-03925-9

**Published:** 2024-06-20

**Authors:** Ke Zhang, Zhongyu Yuan, Shengwei Wang, Shifeng Zhao, Hao Cui, Yongqiang Lai

**Affiliations:** 1 Department of Cardiovascular Surgery, Beijing Anzhen Hospital, Capital Medical University, No.2 Anzhen Road, Chaoyang District, Chaoyang District, Box: 100011, Beijing, China; 2 Beijing Anzhen Hospital, The Key Laboratory of Remodeling-Related Cardiovascular Diseases, Beijing Institute of Heart, Lung and Blood Vessel Diseases, Capital Medical University, Ministry of Education, Beijing, 100029 China; 3 Department of Cardiology, Beijing Anzhen Hospital, Capital Medical University, Beijing, China

**Keywords:** Free fatty acid, Hypertrophic cardiomyopathy, Energy deficiency, Single-center retrospective observational study

## Abstract

**Background:**

Previous studies have shown the importance of energy deficiency and malfunctioning mitochondria in the pathophysiology of hypertrophic cardiomyopathy (HCM). There has been a little research into the relationship between plasma free fatty acids (FFA), one of the heart’s main energy sources, and HCM. We evaluated its clinical importance in HCM to see if there was a link between plasma FFA metabolism and HCM.

**Methods:**

In a single-center retrospective observational study, we investigated 420 HCM patients diagnosed at Beijing Anzhen Hospital between January 1, 2018, and December 31, 2022. Meanwhile, 1372 individuals without HCM (non-HCM) were recruited. 391 non-HCM patients were chosen as controls via a propensity score matching (PSM) study with a 1:1 ratio.

**Results:**

FFA in HCM patients showed statistically significant correlations with creatinine (*r* = 0.115, *p* = 0.023), estimated GFR (*r*=-0.130, *p* = 0.010), BNP (*r* = 0.152, *p* = 0.007), LVEF (*r*=-0.227, *p* < 0.001), LVFS (*r*=-0.160, *p* = 0.002), and LAD (*r* = 0.112, *p* = 0.028). Higher FFA levels were found in HCM patients who had atrial fibrillation and NYHY functional classes III or IV (*p* = 0.015 and *p* = 0.022, respectively). In HCM patients, multiple linear regression analysis revealed that BNP and LVEF had independent relationships with increasing FFA (Standardized = 0.139, *p* = 0.013 and =-0.196, *p* < 0.001, respectively).

**Conclusions:**

Among HCM patients, the plasma FFA concentration was lower, and those with AF and NYHY functional class III or IV had higher FFA levels, and LVEF and BNP were independently associated with increasing FFA. The findings of the study should help inspire future efforts to better understand how energy deficiency contributes to hypertrophic cardiomyopathy (HCM) development.

## Background

There are more than 1400 mutations in at least 11 of the genes producing cardiac sarcomere, which results in hypertrophic cardiomyopathy, a 2‰ percent prevalence of a typical genetic cardiovascular disease among the overall population. Asymmetric left ventricular thickness, fibrosis, and reduced diastolic function are its hallmarks [1–3]. . Pathogenic variants of the sarcomere gene can cause high dynamic contraction, impaired relaxation, and increased cardiac energy consumption, which are all characteristics of hypertrophic cardiomyopathy and increase the risk of arrhythmia, heart failure, and even sudden death [4, 5].

Previous studies have shown that inefficient energy use caused by sarcomere mutations plays a key role in HCM [6]. Free fatty acids produced during fat decomposition are the main energy substrates for healthy myocardium, in which FFA oxidation provides about 70% of energy consumption [7, 8]. Rising plasma and myocardial lipid levels are associated with both an increase in the risk of sudden cardiac death (SCD) [9] and the progression of heart failure (HF) [10–12]. Increased lipolysis brought on by a rise in catecholamines and natriuretic peptides results in elevated FFA levels in individuals with heart failure, which can damage the serum membrane and disrupt the ion channels of heart muscle cells, which helps to explain their clinically demonstrated arrhythmogenic potential [13, 14]. In addition, a number of studies have suggested that pathologic hypertrophy and downregulation of FFA oxidation are related [15–18]. FFA might therefore be crucial in the pathogenesis of HCM. Nevertheless, the relationships between FFA and cardiac morphological and functional features have only been briefly studied. Therefore, we were interested in investigating the association between plasma FFA metabolism and the severity of HCM.

## Methods

### Study population

From January 1, 2018, to December 31, 2022, patients with hypertrophic cardiomyopathy who were continually hospitalized at Beijing Anzhen Hospital were included in this study. The diagnostic criterion of this disease was that the maximum left ventricular wall thickness measured by echocardiography was more than or equal to 15 mm, and no other heart or systemic disease can cause this degree of hypertrophy. Excluding severe coronary disease (coronary angiography demonstrating > 70% stenosis in the epicardial coronary arteries, prior myocardial infarction, coronary artery bypass grafting, or percutaneous coronary intervention), valvular disease, congestive heart failure, connective tissue disease, liver disease, severe renal disease (glomerular filtration rate / EGFR < 30 ml/ (min ·1.73m^2^)), use of anti-inflammatory medicines recently, pregnancy and/or incomplete clinical data. 420 patients with HCM in total were included in this study. As a control, we chose 1372 individuals with complete clinical data who did not have HCM (non-HCM). According to age, gender, BMI, hypertension, hyperlipidemia, and diabetes mellitus, HCM patients were propensity score matched to individuals without HCM. 391 patient pairs were entered after PSM, and in each case, patient demographics and clinical traits were recorded.

The study was approved by the Ethics Committee of Beijing Anzhen Hospital. Patients’ anonymized information will be published in this article with their written informed consent.

### Echocardiography

One to three days after being admitted to the hospital, trained echocardiologists measured the hearts of all patients using transthoracic echocardiography using an ultrasonic device (PHILIP IE33). The interventricular septum (IVS) and ventricular wall thicknesses were measured during diastole. Along with the maximum thickness, it was discovered that the IVS’s representative thickness, which is typically 25 mm below the right coronary sinus nadir, also serves as a proxy for overall thickness. The largest value of the anteroposterior diameter in cardiac cycles was used to express the cardiac chamber diameters through the parasternal left ventricular long-axis segment. To assess the left ventricular ejection fraction and obtain more detailed information, the American Society of Echocardiography’s recommendations were adhered to.

### Laboratory measurements

The concentrations of free fatty acid(FFA), lipoprotein(a) (LPa), total cholesterol (TC), triglycerides (TG), high-density lipoprotein cholesterol (HDL-C), low-density lipoprotein cholesterol (LDL-C), non-high-density lipoprotein cholesterol (non-HDL), creatine (Cr), alkaline phosphatase (ALP), γ-glutamyl transferase (GGT), serum hemoglobin A1c (HbA1c) and effective glomerular filtration rate (eGFR) in the first fasting blood samples collected during hospitalization, which were obtained after a night of fasting, were measured in the Central Laboratory of Beijing Anzhen Hospital, Beijing, China. Brain natriuretic peptide (BNP) and cardiac troponin (cTn) were assessed based on the levels upon admission. Using the Fried Ewald equation, the amount of low-density lipoprotein (LDL) cholesterol was calculated. The Cockcroft-Gault formula was used to calculate creatinine clearance. The collected samples were subjected to analysis within a period of 4 to 6 h, utilizing the Beckman AU5400 (US) automated biochemical analyzer, with the aim of evaluating lipid parameters and other indices. Further blood analyses were conducted with the Sysmex XE-2100, in strict adherence to the manufacturer’s stipulated guidelines. These parameters were assessed using a biochemical analyzer (Hitachi-7600, Tokyo, Japan) utilizing blind quality control samples. For intra- and inter-assays, the corresponding coefficients of variation (CV) were 5% and 10%, respectively.

### Personal measurements

Standard questionnaires were used to acquire information on demographics, lifestyle, medical history, and history of medication. Blood pressure levels of 140/90 mmHg or higher, as well as the usage of antihypertensive medicines, were considered to be diagnosed as hypertension. The most recent recommendations [19] are used to diagnose diabetes mellitus. Having total cholesterol above 200 mg/dL, triglycerides above 150 mg/dL, LDL cholesterol above 130 mg/dL, HDL cholesterol below 40 mg/dL, and/or utilizing lipid-lowering drugs were all defined as having hyperlipidemia, also known as dyslipidemia.

### Statistics analysis

For statistical analysis, SPSS 20.0 was employed. Counts (percentages) were used to represent categorical variables. Mean SD was used to represent continuous data with normal distributions, while median (interquartile range) was used to present continuous variables with non-normal distributions. The chi-square test was used to examine the distribution of genders. The t-test was used to compare the differences between the two groups of measurement data that were normally distributed, and the Mann-Whitney U test was used to test the differences between the two groups of measurement data that had a non-normal distribution. If the data adhered to a normal distribution, Pearson correlation analysis was performed for the association between serum FFA and cardiac anatomical and functional parameters; if the data did not conform to a normal distribution, rank correlation analysis was utilized. The independent variables connected to HCM were found using binary logistic regression analysis. A statistically significant result was determined to be *p* < 0.05 for all two-sided tests.

## Results

A total of 420 patients diagnosed with HCM and 1372 patients without HCM were enrolled. All of them underwent transthoracic echocardiography for the diagnosis of cardiac hypertrophy and cardiac structural and functional parameters, as well as laboratory measurements for metabolic indexes of free fatty acids. Table 1 displays the demographic and clinical traits of the patients in the pre-match and post-match models. After PSM analysis, 391 patients were matched. Table 1 reports the differences in demographic and clinical characteristics variables between before and after matching. The results indicate that the HCM group and control groups are significantly different before matching, while they are statistically indistinguishable after matching, suggesting the efficiency of PSM approach.

**Table 1:** Demographic and clinical characteristics of enrolled patients before and after PSM matching

Variable	Before PSM matching			After PSM matching		
	HCM group (*n* = 420)	Control group (*n* = 1372)	*P*	HCM group (*n* = 391)	Control group (*n* = 391)	*P*
Age (years)	55.78 ± 13.10	58.51 ± 9.89	<0.001	57.27 ± 11.85	58.73 ± 9.95	0.063
Gender (male, n%)	255 (60.7%)	712 (51.9%)	0.002	227 (58.1%)	223 (57.0%)	0.828
Hypertension, n (%)	185 (44.0%)	782 (57.0%)	<0.001	184 (47.1%)	196 (50.1%)	0.431
Hyperlipidemia, n (%)	90 (21.4%)	921 (67.1%)	<0.001	90 (23.0%)	90 (23.0%)	1.000
Diabetes mellitus, n (%)	64 (15.2%)	299 (20.6%)	0.004	63 (16.1%)	76 (19.4%)	0.262
BMI (kg/m^2^)	25.64 ± 3.38	25.90 ± 3.45	0.170	25.78 ± 3.31	25.90 ± 3.73	0.609

PSM: propensity score matching, HCM: Hypertrophic cardiomyopathy, BMI: Body mass index

The baseline characteristics of the population included in the study after PSM are shown in Table 2. The HCM group had higher rates of overall cardiac complications (include coronary heart disease, ventricular tachycardia, atrial fibrillation and NYHY functional class III or IV). Almost all heart anatomical and functional characteristics assessed by transthoracic echocardiography showed highly notable variations (*p*<0.001), and cardiac medication histories included beta blockers and Ca^2+^ channel blockers(CCBs), fitting the basic understandings of this disease. And let’s focus on the serum lipid metabolism index. The FFA (0.43 ± 0.22 vs. 0.47 ± 0.24 mmol/L, *p* = 0.003) and HDL-C (1.10 ± 0.27 vs. 1.18 ± 0.27 mmol/L, *p*<0.001) level were significantly lower than the patients without HCM while LDL-C (2.69 ± 0.84 vs. 2.52 ± 0.87 mmol/L, *p* = 0.002) and nonHDL (3.35 ± 1.02 vs. 2.84 ± 0.88 mmol/L, *p*<0.001) level were significantly higher in patients with HCM than control group. Fasting plasma glucose (5.71 ± 1.98 vs. 5.99 ± 1.71 mmol/L, *p* = 0.039) also were lower than the patients without HCM. And there were strongly significant differences (*p*<0.001) in creatinine (75.95 ± 22.34 vs. 65.98 ± 16.36mmol/L), estimated GFR (90.14 ± 18.50 vs. 95.39 ± 14.48 mL/min per 1.73m2) and BNP (703.13 ± 795.71 vs. 48.32 ± 77.26 pg/mL). To investigate independent factors related with HCM, FFA, nonHDL, and BNP were included into a multivariate binary logistic regression analysis.

**Table 2:** Baseline characteristics of the study enrolled population

Variable	HCM group (*n* = 391)	Control group (*n* = 391)	*P* value	
Coronary heart disease, n (%)	91 (23.27)	2 (0.51)	<0.001	*
Ventricular tachycardia, n (%)	16 (4.09)	1 (0.25)	<0.001	*
Atrial fibrillation, n (%)	81 (20.71)	16 (4.09)	<0.001	*
NYHY functional class III or IV	80 (20.46)	2 (0.51)	<0.001	*
β-Blockers, n (%)	231 (59.08)	118 (30.18)	<0.001	*
CCBs, n (%)	146 (37.34)	7 (1.79)	<0.001	*
Aorta (mm)	32.67 ± 4.10	32.48 ± 3.66	0.501	
Left atrium diameter (mm)	43.46 ± 6.41	35.21 ± 4.51	<0.001	*
IVST (mm)	19.28 ± 4.27	9.62 ± 1.41	<0.001	*
LVEDD (mm)	43.60 ± 6.33	46.44 ± 4.39	<0.001	*
LVPWT (mm)	12.50 ± 2.97	9.05 ± 1.14	<0.001	*
LVM (g)	291.12 ± 91.11	148.02 ± 35.98	<0.001	*
LVM index (g/m2)	166.89 ± 52.10	84.59 ± 20.85	<0.001	*
LVEF (%)	65.11 ± 7.64	64.86 ± 5.64	0.610	
LVFS (%)	36.73 ± 5.05	35.78 ± 3.48	0.003	*
Moderate or severe MR	338 (84.45)	3 (0.77)	<0.001	*
FFA (mmol/L)	0.43 ± 0.22	0.47 ± 0.24	0.003	*
Triglyceride (mmol/L)	1.61 ± 1.01	1.71 ± 1.65	0.279	
Total cholesterol (mmol/L)	4.43 ± 1.01	4.29 ± 1.10	0.070	
HDL-C (mmol/L)	1.10 ± 0.27	1.18 ± 0.27	<0.001	*
LDL-C (mmol/L)	2.69 ± 0.84	2.50 ± 0.87	0.002	*
sdLDL (mmol/L)	0.75 ± 0.38	0.74 ± 0.35	0.726	
nonHDL (mmol/L)	3.33 ± 1.02	2.84 ± 0.88	<0.001	*
Lp (a) (g/L)	0.18 ± 0.24	0.19 ± 0.25	0.793	
Fasting plasma glucose (mmol/L)	5.71 ± 1.98	5.99 ± 1.71	0.039	*
Glycosylated hemoglobin (%)	6.24 ± 1.41	6.11 ± 0.86	0.283	
Creatinine (mmol/L)	75.95 ± 22.34	65.98 ± 16.36	<0.001	*
Estimated GFR (mL/min per 1.73m^2^)	90.14 ± 18.50	95.39 ± 14.48	<0.001	*
BNP (pg/mL)	703.13 ± 795.71	48.32 ± 77.26	<0.001	*
Hs-CRP (mg/L)	1.99 ± 3.07	2.15 ± 3.32	0.494	

HCM: Hypertrophic cardiomyopathy, NYHY: New York Heart Association, CCBs: Calcium channel blockers, IVST: Left ventricular end-diastolic dimension, LVEDD: Left ventricular end-diastolic diameter, LVPWT: Left ventricular posterior wall thickness, LVM: Left ventricular mass, LVEF: Left ventricular ejection fraction, LVFS: Left ventricular shortening fraction, FFA: Free fatty acids, HDL-C: high-density lipoprotein cholesterol, LDL-C: low-density lipoprotein cholesterol, sdLDL: mall dense low-density lipoprotein cholesterol, Lp(a): Lipoprotein a, BNP: Brain natriuretic peptide, Hs-CRP: High-sensitivity C-reactive protein

**Fig. 1:**
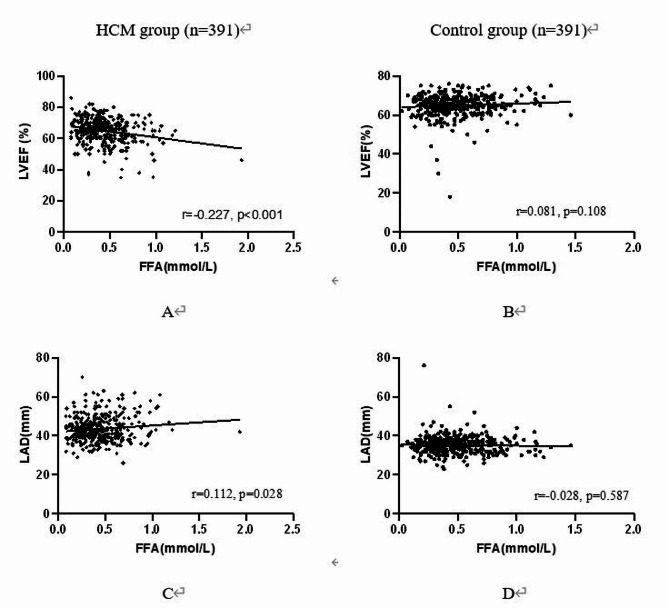
Differences between HCM and control group in the correlation of FFA with EF and left atrium diameter (LAD). **A**, **C** The association of FFA with EF and LAD in HCM patients. **B**, **D** The association of FFA with EF and LAD in control group

In patients with and without HCM, Table 3 presents the plasma FFA levels in association to the clinical characteristics. Patients with HCM who had atrial fibrillation (*p* = 0.015) and NYHY functional class III or IV (*p* = 0.022) had higher FFA levels. Table 4 shows the outcomes of a univariate study of the connection between FFA serum and factors in patients with and without HCM. FFA in HCM patients was positively correlated with creatinine (*r* = 0.115, *p* = 0.023), estimated GFR (*r*=-0.130, *p* = 0.010) and BNP (*r* = 0.152, *p* = 0.007). Meanwhile, there were strong relationships between FFA and LVEF (*r*=-0.227, *p* < 0.001; Fig. 1a), Left atrium diameter (*r* = 0.112, *p* = 0.028; Fig. 1b) and LVFS (*r*=-0.160, *p* = 0.002). With the exception of a marginal link between FFA and BNP (*r*=-0.112, *p* = 0.053), none of these characteristics were significantly associated to FFA in patients without HCM. Following that, using the method of multiple linear regression, independent factors affecting FFA degrees in HCM patients were identified (Table 5). An increase in FFA was independently correlated with LVEF (Standardized =-0.196, p0.001) and BNP (Standardized = 0.139, *p* = 0.013).

**Table 3:** Plasma FFA levels with respect to clinical characteristics of patients with and without HCM

Variables	HCM group (*n* = 391)			Control group (*n* = 391)		
	Present	Absent	*P* value	Present	Absent	*P* value
Coronary heart disease	0.47 ± 0.22	0.42 ± 0.22	0.060	0.50 ± 0.16	0.48 ± 0.24	0.890
Ventricular tachycardia	0.47 ± 0.23	0.43 ± 0.22	0.443	0.230*	0.48 ± 0.24	0.294
Atrial fibrillation	0.49 ± 0.24	0.42 ± 0.22	0.015	0.39 ± 0.16	0.49 ± 0.24	0.115
NYHY functional class III or IV	0.48 ± 0.29	0.42 ± 0.20	0.022	0.41 ± 0.04	0.48 ± 0.24	0.652
β-Blockers	0.44 ± 0.22	0.40 ± 0.20	0.170	0.49 ± 0.23	0.48 ± 0.24	0.600
CCBs	0.42 ± 0.20	0.44 ± 0.24	0.417	0.49 ± 0.14	0.48 ± 0.24	0.934

*n = 1

HCM: Hypertrophic cardiomyopathy, NYHY: New York Heart Association, CCBs: Calcium channel blockers

**Table 4:** Univariate analysis of correlation between variables and FFA in patients with and without HCM

Variables	HCM group (*n* = 391)		Control group (*n* = 391)	
	Correlation coefficient (r)	*P*-value	Correlation coefficient (r)	*P*-value
Age(years)	0.087	0.087	0.023	0.645
BMI (kg/m^2^)	0.035	0.495	0.098	0.052
LVEF (%)	-0.227	< 0.001	0.081	0.108
LVFS (%)	-0.160	0.002	0.069	0.181
LVM index (g/m2)	-0.025	0.624	-0.011	0.829
IVST (mm)	0.038	0.459	0.080	0.113
Left atrium diameter(mm)	0.112	0.028	-0.028	0.587
LVEDD (mm)	0.028	0.580	-0.057	0.260
LVPWT (mm)	-0.046	0.365	0.076	0.134
LVM (g)	-0.017	0.736	0.017	0.738
HDL-C (mmol/L)	-0.042	0.405	0.181	0.000
LDL-C (mmol/L)	0.027	0.598	0.013	0.797
nonHDL (mmol/L)	0.017	0.780	0.040	0.618
Lp (a) (g/L)	0.001	0.991	-0.077	0.131
Fasting plasma glucose (mmol/L)	0.035	0.494	0.085	0.094
Creatinine (mmol/L)	0.115	0.023	0.054	0.285
Estimated GFR (mL/min per 1.73m2)	-0.130	0.010	-0.070	0.165
BNP (pg/mL)	0.152	0.007	-0.112	0.053
Hs-CRP (mg/L)	0.071	0.175	0.098	0.052

HCM: Hypertrophic cardiomyopathy, BMI: Body mass index, IVST: Left ventricular end-diastolic dimension, LVEDD: Left ventricular end-diastolic diameter, LVPWT: Left ventricular posterior wall thickness, LVM: Left ventricular mass, LVEF: Left ventricular ejection fraction, LVFS: Left ventricular shortening fraction, FFA: Free fatty acids, HDL-C: high-density lipoprotein cholesterol, LDL-C: low-density lipoprotein cholesterol, sdLDL: mall dense low-density lipoprotein cholesterol, Lp(a): Lipoprotein a, BNP: Brain natriuretic peptide, Hs-CRP: High-sensitivity C-reactive protein

**Table 5:** Multiple linear regression analysis for the association between variables and FFA in HCM patients

	Standardized coefficients (β)	*P*-value	Tolerance	VIF
LVEF	-0.196	< 0.001	0.996	1.004
BNP	0.139	0.013	0.996	1.004

Multiple = 0.247, R2 = 0.061 F = 10.010, D-W = 1.980

## Discussion

HCM mutations most frequently increase ATP usage and force production at the cellular level, increasing energy requirements [20]. FFA is the heart’s primary energy substrate, which should normally initially accompany impaired energy supply in HCM. Few studies, meanwhile, have been done to clarify how plasma FFA levels and HCM are related. In contrast to patients without HCM, our investigation indicated that the plasma FFA concentration was lower, LVEF and BNP were independently related with rising plasma FFA levels in HCM patients. Additionally, FFA measurements were greater in patients with atrial fibrillation and NYHY functional classes III or IV, indicating a deleterious impact of FFA accumulation in HCM patients.

Maintaining the continuous contraction and relaxation of the heart is a highly complex process that requires a significant amount of energy. The heart’s primary sources of metabolic energy are fatty acid oxidation (FAO) and glucose oxidation, with FAO accounting for 70-90% of the energy supply and the remaining 10-30% coming from glucose and other metabolic substrates [21]. However, when producing the same amount of ATP, FA has a higher oxygen consumption rate than glucose, and its productivity efficiency is lower [22]. This metabolic competition between glucose and FA is known as the glucose-fatty acid cycle (Randle cycle) [23], which helps to maintain energy homeostasis. Interestingly, during cardiac development, the preference for metabolic fuels shifts from fetal glycolysis to adult heart FAO [24, 25]. Patients with HCM have been found to have lower heart FAO rates and higher glucose oxidation compared to healthy hearts [26–28]. Our study found a decrease in plasma FFA concentration in HCM patients, which supports previous research that has linked reduced plasma FFA concentration to the enhancement of pyruvate oxidation, a decrease in fatty acid β oxidation, and a decrease in the PCr/ATP ratio [29, 30]. HCM-associated sarcomeric mutations primarily result in increased ATP usage for the force generation process in the early stages of the disease [31–33]. As cellular hypertrophy and myocardial fibrosis advance, there is an interruption in microcirculation which gives way to myocardial cell hypoxia. With the progress of cell hypertrophy and myocardial fibrosis, microcirculation disturbance leads to myocardial cell hypoxia, which may further promote Randle Shift towards glucose metabolism with higher oxygen utilization rate, and this shift further aggravates energy depletion. This clinical evidence further strengthens the connection between cardiac energy changes and disease phenotype development.

Activation of the sympathetic nervous system can rapidly increase circulating FFA concentrations, primarily due to stimulation of hormone-sensitive lipase activity in adipose tissue by β-adrenoceptor [34, 35]. Besides, the elevation in circulating FFAs significantly impacts the uptake and β-oxidation of fatty acids in the heart. However, studies suggest that impaired β-adrenergic signaling is a common feature in HCM [36–39]. Hence, We speculate that lower levels of circulating FFA in HCM patients may be caused by disturbed β-AR signaling, leading to perturbed PKA-phosphorylation of its targets, although there is limited knowledge available about the physiological changes currently.

One feature that the different varieties of HCM-causing mutations share is an inefficiency of ATP utilization [40–43]. Functional evaluation supports the energy depletion model, as evidenced by higher metabolic respiration activity, abnormalities in calcium handling, contraction force, and the presence of arrhythmias resulting from the HCM-causing mutation produced by CRISPR/Cas9 editing [44]. ^123^I BMIPP (a radioactive fatty acid analogue) myocardial scintigraphy demonstrated that BMIPP uptake was reduced in HCM, and delayed regional BMIPP uptake was the most significant factor in predicting regional function in HCM [45, 46]. In a study conducted at a single center with cross-sectional design, showed the results differed slightly. In male HCM patients but not in females [47], LVMI, LAD, and HDL-C were each independently correlated with rising plasma FFA levels. When comparing our results to those of older studies, it is important to note that our investigation revealed that LVEF and BNP exhibited independent associations with the escalation of plasma FFA levels among individuals diagnosed with HCM, as well as those suffering from atrial fibrillation. Likewise, patients classified as NYHA functional class III or IV displayed elevated FFA levels. In conjunction with the aforementioned research, our results propose a significant involvement of FFA in the etiology and severity of HCM.

A high rate of atrial fibrillation (AF) is seen in HCM, which has been linked to increasing heart failure, a loss in functional capacity, and an increased risk of systemic thromboembolism [48, 49]. Since most information comes from experiments on animals, it is still unknown exactly how FFA produce ventricular arrhythmias [50]. A possible mechanism that could increase the risk of ventricular arrhythmia is the synthesis of lysophospholipids from the breakdown of membrane lipids and acylcarnitine from circulating FFA [51]. FFA may also impede the Na^+^, K^+^, and ATPase pump, causing a rise in intracellular Na^+^ and Ca^2+^ [52] that may raise the risk of arrhythmias [53, 54].

Our study does have certain limitations. First and foremost, the study was an observational one, which cannot exclude the effects of unmeasured and undetected confounding variables, such as dietary habits, or other metabolic factors. Second, FFA is one of the major energy substrates for the heart, however, other energy substrates’ effects on the relationship between energy insufficiency and cardiovascular outcomes were not taken into account in our study. Furthermore, our investigation failed to make a comparison between the FFA levels of individuals diagnosed with HCM and those who suffered from other cardiovascular afflictions not involving HCM, such as dilated cardiomyopathy, diabetic cardiomyopathy, thus impeding our ability to conduct a comprehensive analysis of FFA levels among diverse cardiac conditions. Third, it would be interesting to examine plasma FFA levels between those with overt hypertrophic cardiomyopathy and mutation carriers who don’t suffer from left ventricular hypertrophy. Last but not least, relationships can only be established, not causality, as is typical of prospective research with any observable phenomenon. More research and follow-up are necessary to ascertain the impact of plasma FFA on cardiac and the potential prognostic value of vascular events, as increased FFA levels have been shown to serve as a credible indicator of sudden cardiac death in people with a variety of cardiac conditions.

## Conclusions

Among HCM patients, the plasma FFA concentration was lower, and those with AF and NYHY functional class III or IV had higher FFA levels, and LVEF and BNP were independently associated with increasing FFA. The research results will hopefully serve as useful feedback information for improvements for energy deficiency in hypertrophic cardiomyopathy (HCM) pathogenesis work, and require larger-scale studies to confirm its clinical value.

## Data Availability

Data availability all data included in this study are available upon request by contact with the corresponding author.

## Abbreviations

HCM: Hypertrophic cardiomyopathy
FFA: Free fatty acids
PSM: Propensity score matching
SCD: Sudden cardiac death
HF: Heart failure
BNP: Brain natriuretic peptide
EF: Ejection fraction
LAD: Left atrium diameter
IVST: Interventricular septum thickness
LVEDD: Left ventricular end-diastolic dimension
LVPWT: Left ventricular posterior wall thickness
LVM: Left ventricular mass
